# The impact of aging and physical training on angiogenesis in the musculoskeletal system

**DOI:** 10.7717/peerj.14228

**Published:** 2022-11-03

**Authors:** Magdalena Zmudzka, Jerzy A. Zoladz, Joanna Majerczak

**Affiliations:** Chair of Exercise Physiology and Muscle Bioenergetics, Faculty of Health Sciences, Jagiellonian University Medical College, Krakow, Poland

**Keywords:** Angiogenesis, Physical training, Muscle-bone crosstalk, Skeletal muscle aging, Bone aging, Nitric oxide, VEGF, Capillaries

## Abstract

Angiogenesis is the physiological process of capillary growth. It is strictly regulated by the balanced activity of agents that promote the formation of capillaries (pro-angiogenic factors) on the one hand and inhibit their growth on the other hand (anti-angiogenic factors). Capillary rarefaction and insufficient angiogenesis are some of the main causes that limit blood flow during aging, whereas physical training is a potent non-pharmacological method to intensify capillary growth in the musculoskeletal system. The main purpose of this study is to present the current state of knowledge concerning the key signalling molecules implicated in the regulation of skeletal muscle and bone angiogenesis during aging and physical training.

## Introduction

Skeletal muscle and bone, which are linked through the actions of external mechanical forces generated by gravitation and internal loading *via* muscle contractions, create an efficient working system called the muscle–bone unit (Frost & Schönau, 2000). In an average man with a body mass of 70 kg, the bones constitute about 7% of the body mass *i.e.,* 4.9 kg, whereas the muscles constitute about 40% of the body mass *i.e.,* 28 kg (for review, see Duda et al., 2019). The fundamental mechanism underlying skeletal muscle–bone cooperation during physical exercise is the mechanical loading generated by contracting muscles, which exerts serious metabolic stress (Kemmler & von Stengel, 2019) and triggers biochemical and molecular signalling pathways in both muscle and bone (Frost & Schönau, 2000).

To meet the elevated energy demand during exercise, one of the key muscle tissue responses is an increase in blood flow (for review, see Roseguini & Laughlin, 2019), which enables the delivery of oxygen and nutrient substrates to the working muscles. It is well documented that skeletal muscle has the capacity to drastically increase its metabolic rate during exercise when compared to its level at rest, especially in a trained state (for review, see Roseguini & Laughlin, 2019; Zoladz, Szkutnik & Grassi, 2019). At rest, muscle blood flow amounts to ∼5 mL 100 g muscle^−1^ min^−1^ (for review, see Roseguini & Laughlin, 2019), whereas during a single bout of strenuous exercise, involving small muscle groups such as the quadriceps (∼2.5 kg), muscle blood flow increases to ∼250–400 mL min^−1^ 100 g muscle^−1^ (Andersen & Saltin, 1985), depending on the training status of the studied subjects (for review, see Zoladz et al., 2015). This accounts for a 50–80-fold increase above the resting muscle blood flow level and allows the skeletal muscles to increase their oxygen consumption (VO_2_) from about 3 mL O_2_ kg muscle^−1^ min^−1^at rest to more than 600 mL mL O_2_ min^−1^ 100 g muscle^−1^ (Richardson et al., 1995) during strenuous exercise (for review see, Zoladz et al., 2015). This large increase in muscle blood flow during exercise illustrates the fact that working skeletal muscles are the main consumers of cardiac output during exercise (Andersen & Saltin, 1985).

The energetic needs of bone tissue during physical exercise are less understood. Nevertheless, in view of the data from Elia (1992), bone is a low-rate metabolic tissue and it has a lower increase in metabolic rate during exercise when compared to skeletal muscle. Accordingly, femoral bone blood flow at rest in humans amounted to 1.1 ± 0.4 mL 100 g^−1^ min^−1^, which is ∼4–5 times less than the muscle blood flow at rest (Heinonen et al., 2018). Physical exercise significantly increases femoral bone blood flow in humans, but only to 6.3 ± 1.5 mL 100 g^−1^ min^−1^ (Heinonen et al., 2018). This indicates that, although bone blood flow increases ∼5 times during exercise, its maximal level during exercise is still similar to the muscle blood flow at rest and is ∼40–60 times lower than muscle blood flow during maximal exercise.

One of the body’s strategies to increase blood flow to a given tissue, besides the enhancement of the vasodilative mechanisms in a given tissue (Seals et al., 2009), is an intensification of capillary growth, which improves delivery of oxygen, nutrient substrates, hormones and growth factors on the one hand and enhances removing heat and waste products of cell metabolism (carbon dioxide, hydrogen ions and lactate) on the other hand. It is well established that aging attenuates capillary growth (Coggan et al., 1992; Kusumbe, Ramasamy & Adams, 2014). On the other hand, there is evidence that regular physical training intensifies angiogenesis and capillarisation in various tissues, including skeletal muscle, both in young and aged individuals (Zoladz et al., 2005; Gries et al., 2018; Gliemann et al., 2021), leading to an increase in blood flow during exercise (Dominguez et al., 2010; for review, see Roseguini & Laughlin, 2019). It needs to be underlined that the impact of physical activity on angiogenesis in bone has mainly been studied in animal models, and, similarly to skeletal muscle, it has been reported that physical activity enhances bone angiogenesis (Yao et al., 2004) and increases bone blood flow, as well as improving bone integrity (Stabley et al., 2014).

In this review, the basic state of knowledge concerning the crucial signalling molecules implicated in the regulation of skeletal muscle and bone angiogenesis during aging and physical training are presented. This review aims to provide useful information on the impact of aging on muscle and bone health, as well as the role of physical activity in maintaining the health of the vascular system. The review will benefit both, physicians and exercise physiologists.

## Survey Methodology

Literature was obtained using the PubMed, Web of Science and Google Scholar databases, which were surveyed using different combinations of the keywords ‘angiogenesis’, ‘pro-angiogenic factors’, ‘anti-angiogenic factors’, ‘capillaries’, ‘skeletal muscle’, ‘bone’, ‘aging’, ‘physical training’ or ‘endurance training’, without limits on publication date. Literature reviews and research articles written in English were included, whereas conference abstracts and non-English publications were excluded from the database. The abstracts from the publications in the database were assessed. The criteria for inclusion of a given article were as follows: (1) classical articles reporting crucial discoveries in musculoskeletal angiogenesis; (2) experimental studies demonstrating the impact of physical training on muscle and bone capillarisation in young and older individuals; and (3) experimental studies reporting the impact of physical training on muscle and bone capillarisation obtained from animal models. After pre-selection and applying the above criteria 131 of the most relevant articles, that were consistent with the subject of the study were selected.

### Angiogenesis in skeletal muscle and bone

Angiogenesis is the physiological process of capillary growth. It occurs by sprouting from pre-existing vessels. It is well established that skeletal muscle capillaries may originate *via* two distinct mechanisms, *i.e., via* sprouting or *via* non-sprouting (splitting) angiogenesis, which are induced by several stimuli such as elevated blood flow, muscle contraction and chronic electrostimulation (for review, see Gorski & De Bock, 2019). Capillaries in bone are created by sprouting angiogenesis induced by mechanical loading, *e.g.,* during muscle contraction (Liu et al., 2016). Sprouting angiogenesis includes several stages: an increase in vascular permeability, extracellular matrix (ECM) remodelling, degradation of the basement membrane (BM), proliferation and migration of endothelial cells (ECs) and vessel maturation (Folkman, 1982) (Table 1). In contrast, splitting angiogenesis, also referred to as unorthodox angiogenesis, is based on the repeated insertion of pillar-like structures, without degradation of the BM and endothelial sprouting (Egginton et al., 2001). Nevertheless, each stage of angiogenesis (both in sprouting and splitting) is strictly regulated through a balanced activity series of agents that promote the formation of capillaries (pro-angiogenic factors) and molecules, that inhibit their growth (anti-angiogenic factors) (Table 1).

**Table 1:** Angiogenesis in skeletal muscle and bone: the key pro- and anti-angiogenic factors.

**Stages of angiogenesis**	**Pro-angiogenic factors**	**Anti-angiogenic factors**
Vasodilation and increased vascular permeability	VEGF, Flt-1, KDR, NO^•^, eNOS, HIF-1*α*, PGC-1*α*	TSP-1
Extracellular matrix remodelling and degradation of basement membrane	MMPs (MMP-2, MMP-9, MMP-14)	TIMPs
Endothelial cells proliferation and migration	VEGF, Flt-1, KDR, NO^•^, eNOS, HIF-1*α*, PGC-1*α*	TSP-1
Vessel maturation	VEGF	

**Notes.**

eNOS endothelial nitric oxide synthase Flt-1 fms-like tyrosine kinase receptor (VEGF receptor 1) HIF-1*α* hypoxia-inducible factor-1 subunit *α* KDR fetal liver tyrosine kinase receptor (VEGF receptor 2) MMPs matrix metalloproteinases NO^•^ nitric oxide PGC-1*α* peroxisome proliferator-activated receptor- *γ* coactivator TIMPs tissue inhibitors of metalloproteinases TSP-1 thrombospondin-1 VEGF vascular endothelial growth factor

Based on Folkman (1982); Good et al. (1990); de Vries et al. (1992); Terman et al. (1992); Dvorak et al. (1995); Stamler & Meissner (2001); Tang et al. (2004b); Rullman et al. (2007); Wang et al. (2007); Arany et al. (2008); Leick et al. (2009).

Despite many meaningful discoveries (Table 2), the mechanisms of basal and training-induced capillary growth regulation in the musculoskeletal system is still not fully understood.

**Table 2:** Angiogenesis discovery milestones.

**Year**	**Discovery**	**References**
1794	First observation that new blood vessels originate from pre-existing ones and vascularity is proportional to tissue metabolic requirements	Hunter (1794)
1971	Angiogenesis is found to be necessary in tumour growth and inhibition of angiogenesis can be helpful in anticancer therapy	Folkman (1971)
1980	Discovery of Ca^2+^-dependent endothelial-derived relaxing factor (EDFR), which is now recognised as nitric oxide (NO^•^)	Furchgott & Zawadzki (1980)
1989	Discovery of vascular endothelial growth factor (VEGF) –a key signalling molecule in angiogenesis	Ferrara & Henzel (1989)
1990	Discovery of first endogenous inhibitor of angiogenesis thrombospondin-1 (TSP-1)	Good et al. (1990)
1992	Discovery of hypoxia-inducible factor-1 (HIF-1), the major regulator of skeletal muscle and bone angiogenesis	Semenza & Wang (1992)
1992	Identification of VEGF receptors, *i.e.,* fms-like tyrosine kinase receptor (Flt-1) and fetal liver tyrosine kinase receptor (KDR)	de Vries et al. (1992), Terman et al. (1992)
1993	First observation that inhibition of VEGF decreases density of vessels and suppresses tumour growth *in vivo*	Kim et al. (1993)
2008	Discovery of the mechanism of HIF-independent regulation of exercise-induced skeletal muscle angiogenesis *via* peroxisome proliferator-activated receptor- *γ* coactivator (PGC-1*α*)	Arany et al. (2008)
2014	Discovery of type H and L ECs in bone and the demonstration that type H ECs are involved in bone angiogenesis	Kusumbe, Ramasamy & Adams (2014)

**Notes.**

ECs endothelial cells EDFR Ca^2+^-dependent endothelial-derived relaxing factor Flt-1 fms-like tyrosine kinase receptor (VEGF receptor 1) HIF-1 hypoxia-inducible factor-1 KDR fetal liver tyrosine kinase receptor (VEGF receptor 2) NO^•^ nitric oxide PGC-1*α* peroxisome proliferator-activated receptor- *γ* coactivator TSP-1 thrombospondin-1 VEGF vascular endothelial growth factor

One of the essential stimulators of musculoskeletal capillary growth is endothelium-derived nitric oxide (NO^•^) (Stamler & Meissner, 2001). NO^•^ is synthesised from L-arginine by nitric oxide synthase (NOS). There are three isoforms of NOS: neuronal (nNOS), inducible (iNOS) and endothelial (eNOS). However, in skeletal muscle and bone, capillary growth is mostly implicated with eNOS (Stamler & Meissner, 2001; Milkiewicz et al., 2005; Heinonen et al., 2018). Full activity of NO^•^ is triggered by binding NO^•^ to the heme group of soluble guanylyl cyclase (sGC) and stimulation of cyclic guanosine-3′5′-monophosphate (cGMP) synthesis (for review, see Dulak & Józkowicz, 2003), which in consequence leads to a relaxation of the vascular smooth muscle cells (SMC) and vasodilation.

In addition, NO^•^ contributes to an increase in skeletal muscle and bone angiogenesis through upregulation of the expression of pro-angiogenic factors, including vascular endothelial growth factor (VEGF) (Wang et al., 2004; Milkiewicz et al., 2005). VEGF, initially recognised as the vascular permeability factor (VPF), is one of the most studied critical stimulators of basal and training-induced angiogenesis (Dvorak et al., 1995; Olfert et al., 2010). Its essential role is associated with the promotion of vascular permeability and stimulation of EC proliferation, as well as migration beyond pre-existing vessels (Dvorak et al., 1995). Complete activity of VEGF requires EC membrane-bounded fms-like tyrosine kinase receptors (Flt-1) (receptor 1) (de Vries et al., 1992) or fetal liver tyrosine kinase receptors (KDR) (receptor 2) (Terman et al., 1992). The binding of VEGF to its receptors (Flt-1 and KDR) activates intracellular pathways that control vascular permeability and EC growth. Kim et al. (1993) demonstrated for the first time the significance of VEGF in the promotion of angiogenesis, finding that the inhibition of VEGF decreases the density of vessels and suppress tumour growth *in vivo*. In the musculoskeletal system, VEGF is essential in the preservation of capillary growth (Olfert et al., 2010; Hu & Olsen, 2016). It has been demonstrated in animal models that the inactivation of VEGF gene leads to a ∼64% reduction of muscle capillarity (Tang et al., 2004a), whereas the inhibition of osteoblast-derived VEGF decreases blood vessel density by up to ∼50% in bone (Hu & Olsen, 2016).

The expression of VEGF is modulated by many factors (for review, see Gorski & De Bock, 2019). In the musculoskeletal system, one of the most prominent conditions strictly involved in the regulation of VEGF expression is a local decrease of oxygen level (hypoxia). It is worth mentioning that the strategy of cell adaptation to hypoxia involving angiogenesis has been described by the Nobel Prize winners in Physiology and Medicine (Semenza, Kaelin and Ratcliffe) in (2019). The crucial oxygen sensor, which triggers molecular adaptations to lower levels of oxygen, is hypoxia-inducible factor-1 (HIF-1) (Semenza, 2012), a transcription factor, that contains two subunits: oxygen-dependent (HIF-1*α*) and oxygen-independent (HIF-1*β*).

Under well-oxygenated conditions, HIF-1*α* is bounded by the von Hippel–Lindau protein (pVHL), which recruits the ubiquitin ligase, leading to proteasomal degradation of HIF-1*α* (Wang & Semenza, 1995). On the contrary, when the level of oxygen in tissue decreases, HIF-1*α* is protected from degradation and accumulates in the nucleus, where it associates with HIF-1*β* and binds to short, specific sequences of DNA, called the hypoxia response element (HRE) (Wang & Semenza, 1995; Semenza, 2012).

**Figure 1:**
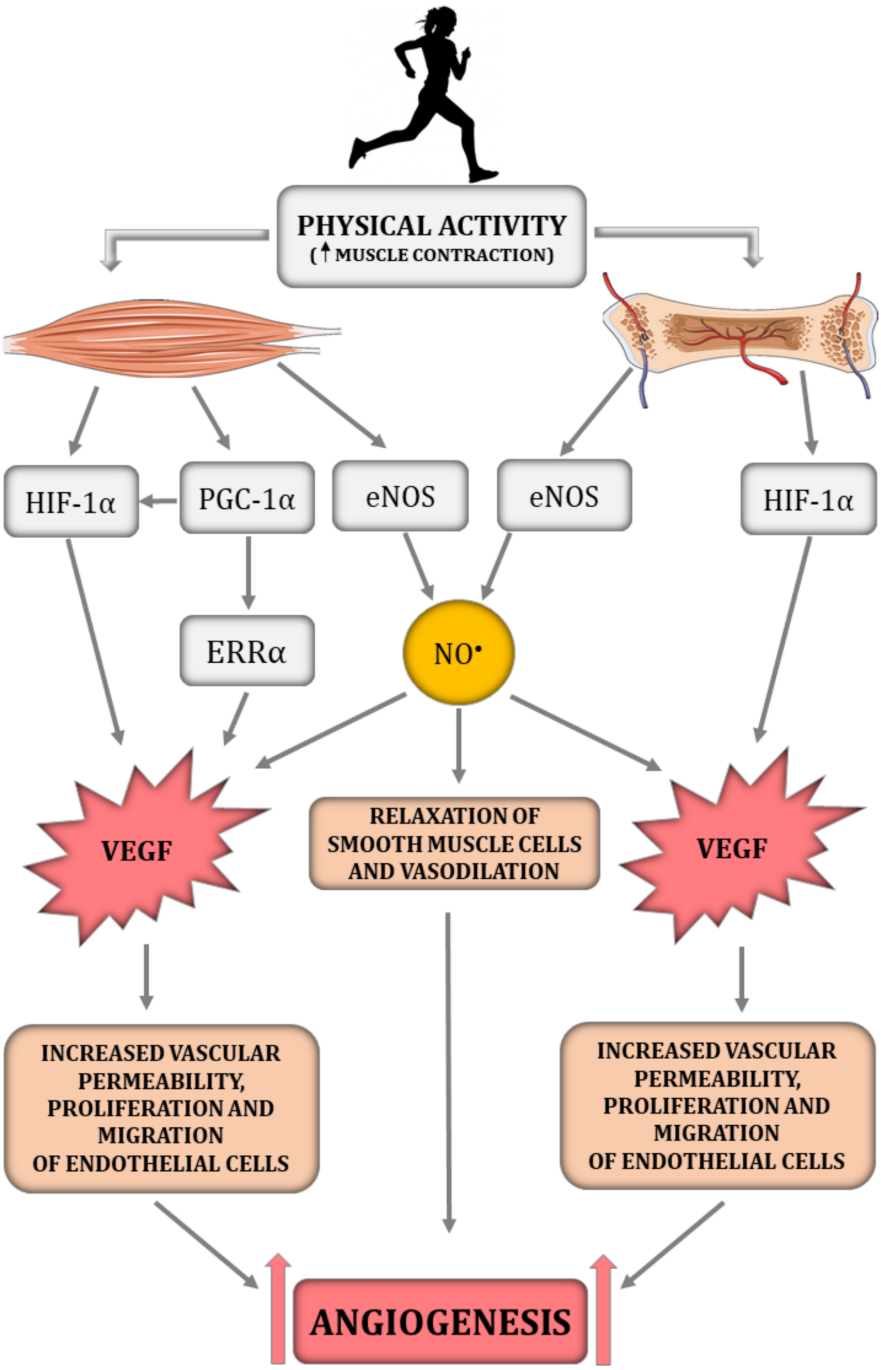
The impact of physical activity on a crucial angiogenesis-related signalling pathways in skeletal muscle–bone unit. eNOS, endothelial nitric oxide synthase; ERR *α*, nuclear estrogen receptor *α*; HIF-1*α*, hypoxia-inducible factor-1 subunit *α*; NO^•^, nitric oxide; PGC-1*α*, peroxisome proliferator-activated receptor-gamma coactivator 1- *α*; VEGF, vascular endothelial growth factor. Figure was created using https://smart.servier.com/.

HIF-1*α* is an essential transcription factor implicated in the regulation of skeletal muscle and bone angiogenesis by upregulating VEGF expression (Fig. 1) (Tang et al., 2004b; Wang et al., 2007). Animal models clearly demonstrate that HIF-1*α* is a crucial molecule involved in bone vascularity (Wang et al., 2007). Mice with deletion of pVHL possess upregulated levels of HIF-1*α* in osteoblasts and thus greater VEGF mRNA expression as well as bone vascularity, while mice with HIF-1*α* deletion in osteoblasts are characterised by a loss of bone vascularity (Wang et al., 2007). It needs to be underlined that, in the musculoskeletal system, NO^•^ bioavailability is implicated in the HIF-1*α*-dependent regulation of VEGF transcription. It has been reported that hyperbaric oxygen treatment increases not only the level of oxygen but also enhances NO^•^ production (Yamamoto et al., 2020). In turn, increased NO^•^ bioavailability decreases prolyl hydroxylase (PHD) activity, the main enzyme participating in HIF-1*α* degradation, thereby leading to HIF-1*α* stabilisation and to an increase in VEGF secretion and intensification of angiogenesis, which was demonstrated in injured rat skeletal muscle (Yamamoto et al., 2020).

Interestingly, previous studies highlighted that, besides the HIF-1*α*-dependent pathway, angiogenesis in skeletal muscle can be regulated by a HIF-1*α*-independent pathway triggered by peroxisome proliferator-activated receptor- *γ* coactivator (PGC-1*α*) (Fig. 1) (Arany et al., 2008; Leick et al., 2009). PGC-1*α* is a transcription factor, that orchestrates mitochondrial biogenesis, oxidative metabolism and transformation of muscle fibres (Arany et al., 2008). It has been demonstrated that PGC-1*α* is upregulated in response to hypoxia (Arany et al., 2008) and physical training (Chinsomboon et al., 2009). PGC-1*α* has also been found to be involved in the HIF-1*α*-independent regulation of VEGF transcription *via* orphan nuclear estrogen-related receptor *α* (ERR *α*) (Arany et al., 2008; Chinsomboon et al., 2009; Leick et al., 2009). In PGC1-*α* knockout mice, muscle VEGF protein content and skeletal muscle capillarisation have been found to be lower by ∼70% and 20%, respectively, compared to control mice (Leick et al., 2009). In addition, 5 weeks of physical training was ineffective in increasing VEGF expression in PGC-1*α* knockout mice, whereas training of control mice led to an increase in VEGF mRNA and VEGF protein expression by ∼60% when compared to control mice (Leick et al., 2009). These results highlight the importance of PGC-1*α* in skeletal muscle angiogenesis, especially after physical training (Leick et al., 2009). The significance of PGC-1*α* in bone angiogenesis has not been demonstrated so far. However the role of PGC-1*α* in bone homeostasis has been recently discussed (for review, see Chen et al., 2022).

It should be added that the expansion of newly formed capillaries beyond pre-existing vessels requires remodelling of the ECM and degradation of BM (Rullman et al., 2007; Rullman et al., 2009). This stage of angiogenesis is regulated by pro-angiogenic matrix metalloproteinases (MMPs) (Tables 1 and 3). MMPs constitute Zn^+2^-dependent endopeptidases, which mediate the degradation of ECM proteins including collagen, a crucial component of the BM. In human skeletal muscles, ECM remodelling is controlled by MMP-2, MMP-9 and MMP-14 (Rullman et al., 2007; Rullman et al., 2009; Hoier et al., 2012), while in bones it is mostly controlled by MMP-2 and MMP-9 (Engsig et al., 2000).

**Table 3:** Pro- and anti-angiogenic factors in the musculoskeletal system.

	**Molecule**	**Role in angiogenesis**	**References**
Pro-angiogenic factors	VEGF	Increases vascular permeability, proliferation and migration of ECs through Flt-1 or KDR binding	Dvorak et al. (1995), Olfert et al. (2010), Hu & Olsen (2016)
	Flt-1	Promotes vascular permeability, proliferation and migration of ECs by binding VEGF	de Vries et al. (1992)
	KDR	Promotes vascular permeability, proliferation and migration of ECs by binding VEGF	Terman et al. (1992)
	HIF-1*α*	Promotes angiogenesis through upregulation VEGF expression	Tang et al. (2004a), Tang et al. (2004b), Wang et al. (2007)
	PGC-1*α*	Increases VEGF expression in skeletal muscle leading to improvement of exercise-induced angiogenesis	Leick et al. (2009), Arany et al. (2008)
	eNOS	Promotes vasodilation and increases vascular permeability through synthesis of NO^•^	Stamler & Meissner (2001), Prisby et al. (2007)
	NO^•^	Promotes vasodilation and increases vascular permeability through stabilisation of HIF-1*α* and upregulation VEGF expression	Milkiewicz et al. (2005), Yamamoto et al. (2020)
	MMPs	Promotes ECM remodelling and degradation of BM by proteolysis of ECM components, *i.e.,* collagen	Rullman et al. (2007), Hoier et al. (2012), Engsig et al. (2000)
Anti-angiogenic factors	TSP-1	Inhibits vasodilation, decreases vascular permeability, proliferation and migration of ECs through inhibition of NO^•^ activity *via* inhibition of sGC and cGMP synthesis	Good et al. (1990), Isenberg et al. (2006)
	TIMPs	Reduces the ECM remodelling and degradation of BM through inhibition of MMPs activity	Rullman et al. (2007), Hoier et al. (2012)

**Notes.**

BM basement membrane cGMP cyclic guanosine-3′5′-monophosphate ECM extracellular matrix ECs endothelial cells eNOS endothelial nitric oxide synthase Flt-1 fms-like tyrosine kinase receptor (VEGF receptor 1) HIF-1*α* hypoxia-inducible factor-1 subunit *α* KDR fetal liver tyrosine kinase receptor (VEGF receptor 2) MMPs matrix metalloproteinases NO^•^ nitric oxide PGC-1*α* peroxisome proliferator-activated receptor- *γ* coactivator sGC soluble guanylyl cyclase TIMPs tissue inhibitors of metalloproteinases TSP-1 thrombospondin-1 VEGF vascular endothelial growth factor

Outside of the pro-angiogenic factors that are important in musculoskeletal capillary growth the role of inhibitors in this process should not be neglected. One of the most significant inhibitors of angiogenesis in the musculoskeletal system is thrombospondin-1 (TSP-1). TSP-1 is stored in the ECM and impairs vasodilation and EC proliferation (Good et al., 1990) (Tables 1 and 3). Moreover, TSP-1 is a strong regulator of NO^•^ activity. The major inhibitory effect of TSP-1 is associated with a limitation of NO^•^ activity *via* TSP-1 dependent inhibition of sGC and cGMP production (Isenberg et al., 2006). By contrast, complete deletion of TSP-1 is accompanied by an increase in iNOS expression in bone (Amend et al., 2015) and upregulation in VEGF expression in skeletal muscle, leading to an improvement of angiogenesis (Malek & Olfert, 2009). Equally important anti-angiogenic factors are tissue inhibitors of metalloproteinases (TIMPs), which are stored in ECM and limit the activity of MMPs, which in consequence impair ECM remodelling and capillary growth (Rullman et al., 2007; Hoier et al., 2012; Chen et al., 2021).

### The impact of aging on skeletal muscle angiogenesis and capillarity

Age-related skeletal muscle mass loss (sarcopenia), is accompanied by a concomitant reduction in muscle strength (dynapenia) (for review, see Degens, 2019; Bilski et al., 2022). Aging in skeletal muscle is progressive and does not initially limit daily human performance; however, after the age of 50, muscle mass is reduced by ∼25–35% when compared to younger people and by the age of 80 it is reduced by ∼40% when compared to young individuals (Lexell, 1995; Janssen et al., 2000). Such a reduction in muscle mass has a strongly negative impact on locomotor performance in elderly people (Degens, 2019). As mentioned above, the loss of muscle mass is accompanied by a decline in muscle strength. Specifically, after the age of 50, muscle strength declines by ∼15% for a decade, leading to ∼20–40% loss of strength between the ages of 60–85 (Booth, Weeden & Tseng, 1994).

Beyond the reduction in muscle mass and strength, aged skeletal muscles are characterised by limited blood flow. The basal femoral artery blood flow in healthy, sedentary older men (55–75 years old), has been found to be reduced by ∼18% in comparison to younger sedentary individuals (20–35 years old) (Dinenno et al., 2001). Interestingly, even in physically active older men (∼63 years old), the muscle blood flow response during exercise (at the intensity corresponding to ∼65% of VO_2max_) has been found to be reduced by ∼26% compared to young active men (Proctor et al., 1998).

One of the most significant consequences of vascular aging is capillary rarefaction (Table 4), which leads to age-related failure in maintaining adequate blood supply to the tissues and the loss of muscle mass in aged individuals. Coggan et al. (1992) demonstrated that overall muscle capillarisation in aged (∼64 years old) sedentary individuals decreases by ∼25% when compared to young people (∼24 years old). Interestingly, it has been found that aged men (∼65 years old and above) lose capillaries regardless of the muscle fibre type (Ryan et al., 2006; Groen et al., 2014) (Table 4). On the other hand, some reports suggest that age-related reduction in muscle capillarity is specific to the type of fibre and is greater in the fast-twitch (type II) than in the slow-twitch (type I) muscle fibres (Croley et al., 2005; Gavin et al., 2007). Moreover, it seems that age-related reduction in the cross-sectional area of fast-twitch muscle fibres is strictly linked with the loss of capillaries.

**Table 4:** The impact of aging on skeletal muscle capillarity in humans.

	Subjects	CD % of change (capillaries per mm^2^)	C/F ratio % of change (capillaries per fibre)	References
Men	Young *vs* Older	↔	↓ ∼25%	Ryan et al. (2006)
	Young *vs* Older	No data	↓ ∼16% (1.85 *vs* 1.55 for Y and O, respectively)	Groen et al. (2014)
	Young *vs* Older	↔ (331 *vs* 286 for Y and O, respectively)	↔ (1.74 *vs* 1.46 for Y and O, respectively)	Barnouin et al. (2017)
	Young *vs* Older	↓ ∼32% (363 *vs* 248 for Y and O, respectively)	↓ ∼35% (2.3 *vs* 1.5 for Y and O, respectively)	Gries et al. (2018)
Women	Young *vs* Older	↔ (190 *vs* 190 for Y and O, respectively)	↓ ∼12% (0.86 *vs* 0.76 for Y and O, respectively)	Croley et al. (2005)
	Young *vs* Older	↔ (340 *vs* 312 for Y and O, respectively)	↔ (1.44 *vs* 1.08 for Y and O, respectively)	Barnouin et al. (2017)
	Young *vs* Older	↓ ∼26% (377 *vs* 281 for Y and O, respectively)	↓ ∼35% (1.7 *vs* 1.1 for Y and O, respectively)	Gries et al. (2018)

**Notes.**

↔ no significant impact of aging ↓ significantly smaller in older CD capillary density C/F ratio capillary-to-fibre ratio O older Y young

Referring to the study by Croley et al. (2005), a loss of fast-twitch muscle fibres by ∼29% in aged women (∼70 years old) is accompanied by an ∼22% reduction in the capillary-to-fibre ratio whereas the preservation of the cross-sectional area of slow-twitch muscle fibres in aging is linked with a well-maintained number of capillaries. Similarly, Gavin et al. (2007) reported that in older men (∼64 years old), a ∼30% reduction of the cross-sectional area of fast-twitch oxidative muscle fibres (type IIA) and a ∼28% reduction of the fast-twitch glycolytic muscle fibres (type IIX) is accompanied by ∼19% and ∼8% decreases in capillary-to-fibre ratio, respectively, whereas in the slow-twitch oxidative muscle fibres (type I) the cross-sectional area and the amount of capillaries are well maintained during aging. It should be highlighted that, in the above-mentioned reports (Ryan et al., 2006; Gavin et al., 2007), type IIB muscle fibre represents type IIX muscle fibre type accordingly to the myosin ATPase stain nomenclature (Smerdu et al., 1994). Despite the fact that a majority of studies demonstrate capillary rarefaction in skeletal muscles in aging, some studies on human and animal models report a surprising increase of muscle capillarity (Davidson et al., 1999; Gavin et al., 2015) or report no impact of aging on muscle capillarity (Rossiter et al., 2005), regardless of the type of muscle fibre. The preservation of muscle capillarisation in aged individuals may be explained by the concurrent loss of muscle fibres and the capillaries surrounding those fibres (Rossiter et al., 2005).

It should be added that age-related attenuation of angiogenesis and capillary rarefaction in the musculoskeletal system is closely associated with decreased level of hormones, especially insulin and sex steroid hormones (estrogens and androgens). It is well established that insulin is a strong stimulator of NO^•^ release (Montagnani et al., 2001) and VEGF secretion (Liu, Petreaca & Martins-Green, 2009). During aging, the production of insulin decreases in a similar manner to the sensitivity of attenuated tissue insulin (Kurauti et al., 2021), hence the loss of insulin signalling is an important age-related cause of angiogenesis impairment. In addition, a lower serum concentration of sex hormones promotes the inflammatory process, increases the risk of cardiovascular disease (CVD) and impairing VEGF-dependent regulation of angiogenesis (Lecce et al., 2014; Grandys et al., 2021). On the other hand, hormone replacement therapy (HRT) *i.e.,* the application of male or female hormones such as estradiol or dihydrotestosterone, has been found to be an effective method of promoting neoangiogenesis after myocardial infarction by increasing the expression of HIF-1*α*, VEGF and the estrogen receptors ER*α* and ER*β* (Chen et al., 2009; Chen et al., 2012). It has also been proven that the application of dihydrotestosterone improves blood flow recovery in the locomotory muscles of older individuals. It has been demonstrated that dihydrotestosterone treatment significantly increases capillary density and enhances muscle blood flow in 24-month-old mice with hindlimb ischemia when compared with age-matched placebo-treated controls (Lam et al., 2019).

The mechanisms of age-related decline in muscle capillarisation are not completely understood; however, one of the key factors underlining capillary rarefaction in the skeletal muscle of older individuals relates to a limited blood flow and attenuation of NO^•^ bioavailability observed both in untrained and trained individuals (Seals et al., 2014; Majerczak et al., 2019). Age-related loss of NO^•^ production is a consequence not only of decreased eNOS activity (Woodman, Price & Laughlin, 2002; Trott et al., 2013), but is also related to an increase in oxidative stress and inflammation with aging (Seals et al., 2014; Majerczak et al., 2019). NO^•^ is a potent activator of early-stage skeletal muscle angiogenesis through the upregulation of a pro-angiogenic VEGF and its receptor, KDR (Milkiewicz et al., 2005). Therefore, it seems that limited NO^•^ activity influences VEGF expression and, consequently, tissue capillarisation during aging. VEGF signalling pathways play an essential role in controlling tissue capillarisation and, as recently demonstrated, an improvement of VEGF signalling is linked to an increased lifespan (Grunewald et al., 2021).

It has been demonstrated that aging downregulates the expression of VEGF mRNA in skeletal muscles by ∼60% (Wagatsuma, 2006; Ryan et al., 2006) and VEGF protein by ∼35% (Croley et al., 2005; Ryan et al., 2006). Age-related decreases in VEGF expression is strictly linked not only to lower NO^•^ bioavailability but also to decreased levels of HIF-1*α*. Specifically, it has been reported that HIF-1*α* mRNA expression is ∼44% lower in aged mice (22-month-old) when compared to young mice (6-month-old) (Wagatsuma, 2006). Similarly, HIF-1*α* protein expression decreases by ∼20% in old rats (20–24-month-old) when compared to young rats (3-month-old) (Yeo, Lim & Ahn, 2020). Little is known about the mechanism of age-related changes of ECM remodelling in skeletal muscle. However, it has been recently demonstrated that aging decreases both the level of pro-angiogenic MMP-2 as well as anti-angiogenic TIMP-2 and TIMP-3 (inhibitors of MMPs), which leads to an impairment of ECM remodelling and increased collagen deposition (Chen et al., 2021). These factors are major causes of attenuated muscle regeneration and increased muscle necrosis in aged individuals (Rahman et al., 2020).

When considering the impact of aging on skeletal muscle capillarisation, one should consider the relationship between muscle capillarisation and the distribution of muscle fibre types. It is well established that type I muscle fibres possess a greater number of capillaries in comparison to fast-twitch muscle fibre (Barnouin et al., 2017). Fast muscle fibres are more susceptible to age-related loss of capillaries when compared to slow muscle fibres (Croley et al., 2005; Gavin et al., 2007). A recent report by Yeo, Lim & Ahn (2020) demonstrated that there are muscle type-dependent differences in the age-related mechanism of angiogenesis. It was found that VEGF expression was downregulated in slow-twitch muscle fibres (soleus) in aging, whereas the expression of VEGF receptor 2 (KDR) was unchanged. In contrast, in aged fast-twitch muscle (EDL), VEGF expression was not altered, while KDR expression was strongly downregulated in slow-twitch muscles. Additionally, HIF-1*α* expression during aging was well maintained in the soleus muscle while significantly decreased in the fast-twitch EDL muscle (Yeo, Lim & Ahn, 2020). Therefore, based on this recent report, the mechanism of age-related attenuation of angiogenesis in varied muscle types needs further examination.

### The impact of aging on bone angiogenesis

A decrease in bone mineral density (BMD) is a typical manifestation of osteoporosis and constitutes a main hallmark of bone aging (Korhonen et al., 2012). Bone fragility, weakness and higher vulnerability to fractures are the main consequences of age-related decrease in BMD (Kanis, 2002), leading to a greater risk of falls and medical complications in elderly individuals (Kemmler & von Stengel, 2018). It has been demonstrated through quantitative computed tomography that the BMD of trabecular bone at central sites decreases in middle aged (40–60 years old) women and men by ∼55% and ∼46%, respectively, but age-related decrease in BMD (∼24% in women and ∼26% in men) remain similar at peripheral sites when compared to young adults (20–29 years old) (Riggs et al., 2004).

One of the crucial factors of age-related bone loss is the limitation of bone blood flow (Ramasamy et al., 2016). It has been found that blood flow in the proximal femur is reduced by ∼49% in females and males at age 80 when compared to younger individuals (20–55 years old) (Lahtinen et al., 1981). Limited local blood flow in aging is correlated with significant reductions of columnar vessels and endothelial bud structures in the proximity of the growth plates (Kusumbe, Ramasamy & Adams, 2014; Ramasamy et al., 2016). Aging gradually decreases the number of type H ECs, which are essential for local bone capillary growth (Kusumbe, Ramasamy & Adams, 2014). Furthermore, the main cause of age-related loss of type H vessels is the limited blood flow, which leads to an attenuation of capillary growth (Ramasamy et al., 2016).

The mechanisms underlying age-related loss of bone capillaries are not well understood; however, it has been demonstrated that, as with skeletal muscle, aging attenuates NO^•^ bioavailability and leads to limited blood flow to bone. Prisby et al. (2007) found that decreased NO^•^ levels in old rats (24–26-month-old) is one of the major causes of age-related impairment of vasodilation and limited blood flow in various parts of the femur. Furthermore, it seems that, as in skeletal muscle, the downregulation of VEGF expression in bone after aging (Ramasamy et al., 2016) is a consequence of reduced HIF-1*α* activity. Older mice (57–70-week-old) are characterised by a decreased expression of HIF-1*α* mRNA in bone when compared to young mice (2–4-week-old); this decrease contributes to the loss of type H ECs, which, as mentioned above, are crucial for bone capillary growth (Kusumbe, Ramasamy & Adams, 2014). Therefore, as with skeletal muscle, aging attenuates angiogenesis in bones, leading to a decrease in bone capillarity.

### The impact of physical training on skeletal muscle and bone capillary growth in aging

Physical activity is a potent non-invasive strategy to prevent non-communicable diseases such as CVD, diabetes or sarco-osteoporosis. But, as a consequence of technological improvement, physical activity is now simply required for the maintenance of general health (Pedersen & Saltin, 2015; Booth et al., 2017; Bettis, Kim & Hamrick, 2018; Li et al., 2019). According to the latest concept of cardiovasomobility, the maintenance of health and quality of life is related to the cardiovascular and skeletal muscle systems, and is linked to physical activity (Trinity et al., 2022). Indeed, many studies demonstrate that lifelong exercise is associated with preserved cardiovascular and musculoskeletal function (Iversen et al., 2011; Fiuza-Luces et al., 2018; Gries et al., 2018; Gliemann et al., 2020), whereas a sedentary lifestyle is accompanied by cardiovascular and skeletal muscle dysfunction that accelerates the onset of varied diseases (Booth et al., 2017). The implementation of physical activity as a daily health requirement seems to be especially important regarding aging, since age is an independent risk factor of CVD (Benjamin et al., 2017).

In the cardiovascular system age-related endothelial dysfunction and arterial stiffness lead to impaired blood flow to the vital organs of the human body, such as the brain, lungs, kidneys, heart and skeletal muscle. One of the hallmarks of age-related endothelial dysfunction is NO^•^ attenuation, which, in the cardiovascular system, is a key vasodilatory and anticoagulatory molecule (Seals et al., 2009). The impact of physical activity on vascular function, besides attenuation of the traditional CVD risk factors (*e.g.,* hyperlipidaemia, hyperglycaemia, obesity or hypertension), is related to an enhancement of NO^•^ bioavailability (Seals et al., 2009; Nyberg et al., 2012; Fiuza-Luces et al., 2018), as well as the integrity of the glycocalyx layer (Majerczak et al., 2017). The enhancement of NO^•^ bioavailability after the physical training is important not only for the cardiovascular system but also for angiogenesis, both in skeletal muscles and bone (Wang et al., 2004; Milkiewicz et al., 2005).

Promotion of capillary growth after physical training in the muscle–bone unit is triggered by mechanical loading, which is generated during repeated muscle contraction and capillary shear stress resulting from increased blood flow during muscle contraction (Frost & Schönau, 2000; Stabley et al., 2014). Specifically, it has been demonstrated on C2C12 myotube cultures that laminar pulsating fluid flow imitating shear stress in blood vessels activates the signalling pathways involved in muscle fibre size adaptation, including insulin growth factor 1 (IGF-1), mechano growth factor (MGF), VEGF, interleukin-6 (IL-6), cyclooxygenase-2 (COX-2) and myostatin (Juffer et al., 2014). In addition, it has been found that shear stress enhances NO^•^ production in a dose-dependent manner. The authors suggest that shear stress applied on the ECM is a crucial for mechanotransduction in muscle and the subsequent activation of signalling pathways involved in muscle adaptation. The suggested mechanism of mechanotransduction is related to the deformation of ECM by shear stress, stimulation of an increase in Ca^2+^ intracellular concentration and activation of the NOS signalling pathway leading to an increase in NO^•^ production and growth factor expression, including pro-angiogenic VEGF (Fig. 1) (Juffer et al., 2014). In addition, the production of NO^•^, which is a key signalling molecule in the angiogenesis depends on the magnitude of shear stress. Specifically, it has been found that activation of eNOS in Human Umbilical Vein Endothelial Cells (HUVECs) is downregulated by 4 dyn/cm^2^ of shear stress but is upregulated by 15 dyn/cm^2^ (Zeng & Liu, 2016).

When considering the impact of physical training on NO^•^ bioavailability, which is a pro-angiogenic factor, it has been demonstrated that 6 weeks of endurance training or sprint interval training increases microvascular eNOS content in skeletal muscle by ∼14 and ∼36%, respectively (Cocks et al., 2013). This result, at the first glance, might suggest that sprint interval training is indeed more effective than a continuous endurance training programme for improvements in eNOS activity; however, this issue requires more and especially longer observations to ascertain the true effect of varied exercise modality on cardiovascular adaptation. Similar to muscle, 10–12 weeks of exercise training significantly increases endothelium-dependent NO^•^-mediated vasodilation of femoral principal nutrient artery (by 16%) and enhances bone blood flow in young rats (4–6-month-old) (Dominguez et al., 2010).

Beyond an increase in NO^•^ bioavailability, physical training increases also other important pro-angiogenic factors *i.e.,* VEGF expression both in skeletal muscle (Gavin et al., 2007; Delavar et al., 2014) and in bone (Yao et al., 2004). It has been reported that 8 weeks of cycling training (at an intensity corresponding to 65% of VO_2max_) increases exercise-induced VEGF mRNA expression in the skeletal muscle of young men by ∼82% (Gavin et al., 2007). An increase in VEGF protein expression after 8 weeks of training has also been demonstrated in an animal model, which was similar in varied muscle types; specifically, VEGF protein expression increased by ∼23% in the soleus, ∼21% in the plantaris, ∼19% in the gastrocnemius and ∼25% in the EDL of mice (Delavar et al., 2014).

The crucial role of the VEGF signalling pathway for training-induced muscle capillary growth has been demonstrated by Olfert et al. (2010). They found that 6 weeks of running in mice lacking VEGF (myocyte-specific VEGF gene deletion) was accompanied by no change in capillarisation, whereas capillary density and capillary-to-fibre ratio increased by 59% and 33%, respectively, in the deep portion of the gastrocnemius muscle in control mice (Olfert et al., 2010).

Other than VEGF, the role of PGC-1*α* or MMPs in training-induced angiogenesis in the skeletal muscles should not be overlooked. As mentioned above, PGC-1*α* orchestrates mitochondrial biogenesis and the transformation of muscle fibres (Arany et al., 2008). Moreover, it is recognized as a strong regulator of exercise-induced capillary growth by upregulation of VEGF expression *via* orphan nuclear receptors (ERR *α*) (Arany et al., 2008) (Fig. 1). It has been found that 2 weeks of voluntary wheel running in rodents increases PGC-1*α* expression and elevates muscle capillary density twofold in skeletal muscle (Chinsomboon et al., 2009). In addition, 5 weeks of physical training was ineffective in increasing VEGF expression in the skeletal muscle of PGC-1*α* knockout mice, whereas, in control mice, training led to an increase in VEGF mRNA and VEGF protein expression by ∼60% (Leick et al., 2009). This result clearly demonstrates the importance of PGC-1*α* in the training-induced increase of VEGF expression in skeletal muscle.

Physical training has also been found to have an impact on ECM remodelling. It has been demonstrated that 5 weeks of one-legged exercise in young men increases skeletal muscle MMP-2 mRNA, MMP-9 mRNA, MMP-14 mRNA and MMP-2 protein expression after 10 days of training, which remain elevated after 5 weeks of training, whereas no training-induced changes in MMP-9 protein expression have been observed (Rullman et al., 2009). Considering the importance of anti-angiogenic factors in capillary growth, it has been demonstrated that two weeks of cycling training has no effect on TSP-1 mRNA and protein expression in human skeletal muscles (Hoier et al., 2020). Similarly, eight weeks of treadmill running (1 h/day, 5 days/week) had no impact on TSP-1 mRNA in the skeletal muscles of rats (Olfert et al., 2006).

Similarly as with the skeletal muscles, physical training significantly improves bone angiogenesis. It has been observed that five weeks of endurance training increases VEGF expression and blood vessels in bone. Specifically, it has been demonstrated that 10 days of training (at an intensity corresponding to 60% of VO_2max_) in young rats (9-week-old) upregulates VEGF expression by ∼150% and its receptor Flt-1 mRNA by ∼80% in the cancellous part of the tibia, whereas in the tibia periosteum, VEGF mRNA expression increases by ∼86% and Flt-1 mRNA by ∼92% (Yao et al., 2004). This is accompanied by an elevation of tibia blood vessels by ∼19% after 2 weeks of training and by ∼26% after 5 weeks of training (Yao et al., 2004). In contrast, blockading of the VEGF protein (through the application of an anti-VEGF antibody) can completely prevent bone vascular adaptation to 5 weeks treadmill training, suggesting that VEGF is a crucial molecule for training-induced bone capillary growth (Yao et al., 2004). Taken together, these results clearly demonstrate that the VEGF signalling pathway is essential for training-induced improvement of capillarity in the skeletal muscle–bone unit. As mentioned above, the role of PGC-1*α* in training-induced angiogenesis in bones has not been described so far.

One of the more intriguing facts about the process of angiogenesis in the musculoskeletal system after physical training is the impact of exercise on the muscle–bone crosstalk. Physiologically close connections between muscle and bone are visible, on the one hand, in age-related sarco-osteoporosis, where reduced physical activity accompanied by a limited amount of muscle contractions (reduction in mechanical loading) induces muscle and bone atrophy (Bettis, Kim & Hamrick, 2018; Kemmler & von Stengel, 2018; Li et al., 2019), and, on the other hand, in physical training in which an increased level of muscle contractions improves both muscle and bone functioning (Kemmler & von Stengel, 2018).

It should be underlined that improvement of the structure and function of the muscle–bone unit after physical training is not only related to mechanical forces (mechanical crosstalk) but also depends on the existence of muscle–bone communication through release of varied soluble factors by the muscles and bones considered as the endocrine organs (biochemical crosstalk). Biochemical crosstalk occurs *via* secretion of skeletal muscle-derived myokines, *i.e.,* nitric oxide, irisin, myostatin, interleukin (IL)-6, IL-7, IL-15, IGF-1, fibroblast growth factor 2 (FGF-2), brain-derived neurotrophic factor (BDNF) and lactate, as well as through bone-derived factors such as osteocalcin, sclerostin, fibroblast growth factor 23 (FGF23), prostaglandin E2 (PGE2), transforming growth factor *β* (TGF-*β*) and Wnts (Karsenty & Mera, 2018; Bonewald, 2019; Chow et al., 2022).

Special attention should be paid to so-called exerkines, which are released by varied tissues (*e.g.,* the heart, muscles, adipose tissue) during exercise. Exerkines might have a potential role in the prevention and treatment of chronic diseases (Chow et al., 2022). For example, bone-derived osteocalcin improves muscle metabolism during exercise by increasing glucose and fatty acids uptake by the skeletal muscles, which is crucial for muscles to adapt to training (Mera et al., 2016). Interestingly, it has been presented in an animal model that aging decreases the secretion of osteocalcin, whereas physical training enhances its levels in the blood (Mera et al., 2016). On the other hand, muscle-derived factors (myokines) such as myostatin (a main inhibitor of skeletal muscle growth and repair) and IL-6 (an energy sensor) influence bone metabolism (Legård & Pedersen, 2019). Specifically, myostatin-null mice display massive muscle hypertrophy and a significantly increased bone mineral density (Bonewald, 2019). In addition, IL-6 increases the production of osteocalcin in bone and regulates bone remodelling (Blanchard et al., 2009).

One should consider that some of the above-mentioned soluble factors, which are important in muscle–bone crosstalk are involved in the process of angiogenesis such as NO^•^, IGF-1 and Wnts (Wang et al., 2004; Milkiewicz et al., 2005; Foulquier et al., 2018; Alcazar et al., 2020; Zhang et al., 2020). Therefore, the improvement of the structure of the muscle–bone unit after physical training, which is of great importance for attenuating age-related sarco-osteoporosis, also seems to be connected with an improvement of muscle–bone communication (Bettis, Kim & Hamrick, 2018; Bonewald, 2019).

When considering the impact of endurance training on skeletal muscle capillarity, it has been demonstrated that long-term (∼8 years of training) endurance-trained young athletes had ∼26% greater capillary density than untrained subjects (245 *vs* 308 capillaries per mm^2^of muscle for untrained and trained athletes, respectively) and ∼11% higher capillary-to-fibre ratio (∼1.9 *vs* 2.1 capillaries per fibre for untrained and trained athletes, respectively) (Zoladz et al., 2005). Experimental studies indicate that capillary growth in young individuals occurs after 4 weeks of continuous cycling training (at an intensity corresponding to ∼60–68% of VO_2max_). Specifically, training elevates capillary density by ∼12% (511 *vs* 571 capillaries per mm^2^ of muscle before and after training, respectively) and capillary-to-fibre ratio by ∼23% (2.47 *vs* 3.03 capillaries per fibre before and after training, respectively) (Hoier et al., 2012). A longer period of training has a less pronounced effect on capillary density. Specifically, after three months of cycling training (at an intensity corresponding to ∼70% of VO_2max_) capillary density increased by ∼17% and capillary-to-fibre ratio by ∼29% (Murias et al., 2011). In addition, it has been reported that even six months of jogging sessions (at an intensity corresponding to ∼75% of VO_2max_) increased capillary density by ∼20% (435 *vs* 520 capillaries per mm^2^ of muscle before and after training, respectively) and capillary-to-fibre ratio by ∼23% (1.42 *vs* 1.75 capillaries per fibre before and after training, respectively) (Baum et al., 2015) (Table 5).

**Table 5:** The impact of endurance training on skeletal muscle capillarity.

	Subjects	Type of training	CD % of change (capillaries per mm^2^)	C/F ratio % of change (capillaries per fibre)	References
Young	Habitually active men	1-month endurance training on cycling ergometer (moderate-intensity)	↑ ∼12% (511 Pre *vs* 571 Post)	↑ ∼23% (2.47 Pre *vs* 3.03 Post)	Hoier et al. (2012)
	Physically active men	3-month endurance training on cycling ergometer (moderate-intensity)	↑ ∼17% (291 Pre *vs* 339 Post)	↑ ∼29% (1.7 Pre *vs* 2.2 Post)	Murias et al. (2011)
	Untrained men	6-month endurance training *i.e.,* jogging (moderate-intensity)	↑ ∼20% (435 Pre *vs* 520 Post)	↑ ∼23% (1.42 Pre *vs* 1.75 Post)	Baum et al. (2015)
	Untrained, physically active (UT) *vs* endurance trained men (ET)	∼8 years endurance training *i.e.,* long distance running, cross country skiing or cycling	↑ ∼26% in ET (245 *vs* 308 for UT and ET, respectively)	↑ ∼11% in ET (1.9 *vs* 2.1 for UT and ET, respectively)	Zoladz et al. (2005)
Older	Physically active men	3-month endurance training on cycling ergometer (moderate-intensity)	↑ ∼28% (336 Pre *vs* 429 Post)	↑ ∼43% (1.4 Pre *vs* 2.0 Post)	Murias et al. (2011)
	Sedentary (Sed) *vs* very active women (vA)	20 years endurance training *i.e.,* running, cycling (moderate or high-intensity)	↑∼15% in vA (356 *vs* 409 for Sed and vA, respectively)	↑ ∼27% in vA (1.30 *vs* 1.65 for Sed and vA, respectively)	Gliemann et al. (2021)
	Untrained *vs* endurance trained men and women	↑20–50 years endurance training *i.e.,* long distance running, cross-country running and cycling	No data	↑∼27% in ET (1.39 *vs* 1.77 for UT and ET, respectively)	Iversen et al. (2011)
	Sedentary (Sed) *vs* physically active (PA) women and men	>50 years aerobic exercises *i.e.,* running, cycling (moderate-intensity)	↑ ∼20% and ∼40% in PA women and men, respectively compared to Sed (281 *vs* 338 for Sed and PA women, respectively; 248 *vs* 347 for Sed and PA men, respectively)	↑ ∼46% and ∼60% in PA women and men, respectively compared to Sed (1.1 *vs* 1.6 for Sed and PA women, respectively; 1.5 *vs* 2.4 for Sed and PA men, respectively)	Gries et al. (2018)

**Notes.**

↑ significantly larger CD capillary density C/F ratio capillary-to-fibre ratio ET endurance trained PA physically active Post after the training Pre before the training Sed sedentary UT untrained, physically active vA very active

It should be underlined that training-induced capillary growth is specific to the fibre type both in young men and women. Namely, 2-months of moderate-intensity cycling in young men (at an intensity corresponding to 65% of VO_2max_) increased capillary density by ∼29%, ∼35%, ∼13% and capillary-to-fibre ratio by ∼32%, ∼25%, ∼21% in type I, IIA and IIB (IIX) muscle fibres, respectively (Gavin et al., 2007). The same training program in young women elevated capillary density by ∼20%, ∼21%, ∼30% and capillary-to-fibre ratio by ∼17%, ∼19%, ∼33% in types I, IIA and IIB (IIX), respectively (Gavin et al., 2015). Interestingly, it is suggested that training-induced angiogenesis (an increase in VEGF protein content, capillary density and capillary-to-fibre ratio) precedes fibre type transformation from glycolytic (IIB + IID/X muscle fibre types) to more oxidative muscle fibres (IIA) (Waters et al., 2004).

Physical training stimulates skeletal muscle capillary growth, not only in the young but also in older individuals (Table 5). Gavin et al. (2007) observed that angiogenic response (VEGF mRNA and protein expression) after 8 weeks of exercise training was found to be similar between both young and older individuals, despite the lower basal skeletal muscle capillarisation in older untrained men compared to young men’s skeletal muscle. This suggests that aging does not impair training-induced angiogenesis. Additionally, as described by Murias et al. (2011), cycling training in previously untrained men (aged ∼69 years old) performed at intensity corresponding to ∼70% of VO_2max_ and lasting three months, increased capillary density by ∼28% (336 *vs* 429 capillaries per mm^2^ of muscle before and after training, respectively) and capillary-to-fibre ratio by ∼43% (1.4 *vs* 2.0 capillaries per fibre before and after training, respectively).

Several authors reported that, even in aging, regular endurance training (such as long-distance running or cycling) preserves muscle mass, capillarisation and the oxidative capacity of the musculoskeletal system (Iversen et al., 2011; Gavin et al., 2015). The evident effect of the impact of physical training on the angiogenic response of older individuals is especially visible in aged women and men who have practiced endurance training for many years *i.e.,* for more than two decades (Iversen et al., 2011; Gries et al., 2018). In the study by Iversen et al. (2011), the skeletal muscle of aged individuals was characterised by a ∼27% higher capillary-to-fibre ratio when compared to elderly untrained subjects (1.77 *vs* 1.39 capillaries per fibre for endurance trained and untrained, respectively). Furthermore, as demonstrated by Gries et al. (2018), lifelong exercise (∼52 years) performed for ∼7 h per week and five days of exercise per week in the group of older men and women (∼72–74 years old) may counteract age-related decreases in capillarisation and aerobic enzyme activity. Specifically, it has been found that capillary density in older trained women and men (338 *vs* 347 capillaries per mm^2^ in lifelong exercising women and men, respectively) is similar to that observed in young individuals subjected to training (377 *vs* 363 capillaries per mm^2^ for young, trained women and men, respectively).

Interestingly, as recently presented by Gliemann et al. (2021), in the group of postmenopausal women (61–62 years old), a high activity level (20 years of training consisting of about 3.6 h per week of moderate-intensity physical activity combined with about 2.5 h per week of high-intensity training) was required to augment muscle capillarisation. The greater capillarisation in the group of highly active women was accompanied by a significantly improved leg blood flow and leg oxygen uptake during exercise (Gliemann et al., 2021).

The summary of the effects of exercise training on skeletal muscle capillarisation in healthy subjects has been recently presented in a meta-analysis by Liu et al. (2022). Based on 57 trials from 38 studies, they demonstrated that there was a 21% higher relative change in the muscle capillary-to-fibre ratio after continuous moderate-intensity training (50–80% of VO_2max_) and 54% increase in this parameter after high-intensity interval training (80–100% of VO_2max_), whereas low-intensity training (<50% of VO_2max_) was less effective (Liu et al., 2022). However, these intriguing observations regarding the capillarisation responses to physical training concern a rather limited period of training (up to 48 weeks for endurance training, and up to a maximum of 8 weeks of interval training), during which more intense training (mainly interval) seems to be more effective than continuous endurance training in terms of its impact on muscle capillarisation.

Furthermore, the conclusions drawn from this meta-analysis—that high-intensity interval training (80–100% of VO_2max_) is more effective than moderate-intensity training (50–80% of VO_2max_)—seems to be in contradiction with the real-life observations in athletes. Namely, athletes who apply chiefly moderate training intensities (long distance runners), when studied after several years of training, possess higher capillary density in their muscles than sprint athletes who trained at much higher intensities (Table 6). Indeed, Torok et al. (1995) found a significant difference in muscle capillarisation between sprinters (with the following maximal running performances: faster than 11.1 s for 100 m, 24 s for 200 m, or 50 s for 400 m) and distance runners (faster than 16 min: 45 s for 5,000 m). Distance runners revealed significantly higher capillary density (∼409 capillaries per mm^2^) and capillary-to-fibre ratio (3.2 capillaries per fibre) than sprinters (323 capillaries per mm^2^ and 2.2 capillaries per fibre) (Torok et al., 1995). The augmented muscle capillarity in the endurance trained athletes (437 capillaries per mm^2^ and 2.8 capillaries per muscle fibre in the legs) has been also demonstrated in a group of elite male Norwegian cross-country skiers (∼11 years of training) (Ørtenblad et al., 2018). Interestingly, there was no significant difference between leg and arm capillarity in this group of subjects (394 capillaries per mm^2^ and 3.0 capillaries per muscle fibre in arm). Furthermore, in the study by Zoladz et al. (2005), it was shown that the capillarisation of the quadricep muscles of sprinters was not higher than in endurance trained athletes (Table 6). Therefore, the above-mentioned studies (Torok et al., 1995; Zoladz et al., 2005) do not support the conclusion offered by the meta-analysis by Liu et al. (2022), that high-intensity interval training is indeed more effective in augmenting muscle capillarisation than continuous endurance training. Hence, the issue of the impact of low/moderate *vs* high-intensity exercise training on capillary density in case of long-lasting training (years) needs to be explored with further studies.

**Table 6:** Skeletal muscle capillarisation in athletes.

References	Subjects (M/F)	Years of training	Skeletal muscle capillarisation			
			Endurance trained		Sprinters	
			CD (capillaries per mm^2^)	C/F ratio (capillaries per fibre)	CD (capillaries per mm^2^)	C/F ratio (capillaries per fibre)
Coyle et al. (1991)	Elite national class cyclist (15/0)	∼9 yr	464	2.90	No data	No data
Torok et al. (1995)	Distance runners (6/0) *vs* sprinters (6/0)	several years	409	3.2	323	2.2
Zoladz et al. (2005)	Endurance trained athletes (9/0) *vs* sprint-power trained athletes (8/0)	∼8 yr ∼13 yr	308	2.1	325	2.1
Ortenblad et al. (2018)	Elite cross-country skiers (10/0)	∼11 yr	Leg: 437 Arm: 394	Leg: 2.8 Arm: 3.0	No data	No data
van der Zwaard et al. (2018)	International road cyclist (14/0) *vs* track sprinters (6/0)	several years	531	2.9	420	2.8

**Notes.**

CD capillary density C/F ratio capillary-to-fibre ratio F female M male yr years

It should be mentioned that, as with young individuals, training-induced capillary growth in elderly people has been found to be fibre type-specific. 8 weeks of cycling training in aged men (at an intensity corresponding to 65% of VO_2max_) increased capillary density by ∼16% in type I and by ∼20% in type IIA muscle fibres. In addition, thecapillary-to-fibre ratio was augmented after this training by ∼19% and ∼20% in type I and IIA muscle fibres, respectively (Gavin et al., 2007). The impact of training on the type IIX muscle fibres capillarisation was not significant. The same training program performed in women elevated capillary density by ∼20%, ∼6%, ∼11% in I, IIA and IIX muscle fibres, respectively, and it increased the capillary-to-fibre ratio by ∼23%, ∼44%, ∼43% in I, IIA and IIX muscle fibres, respectively (Gavin et al., 2015).

Physical training in aged individuals also improves bone microvascularisation. It has been reported that 8 weeks of swimming training is a highly effective strategy for augmenting capillary growth in the femurs of older rats (Viboolvorakul et al., 2009). Swimming elevates bone vascularity by ∼4% in aged rats (20–22-month-old) when compared to sedentary counterparts (∼6.88% *vs* ∼11.25% in aged sedentary and trained rats, respectively) (Viboolvorakul et al., 2009). Nevertheless, there is still few data referring to the impact of physical activity on bone capillary growth in older individuals.

When considering the mechanism underlying training-induced skeletal muscle angiogenesis in the elderly, it seems to be the same as in young individuals. Regular physical activity significantly increases VEGF expression in trained individuals (∼71 years old), even by ∼230% in comparison to untrained subjects at a similar age (Iversen et al., 2011). In addition, physical training in the elderly enhances the expression of PGC-1*α*, which upregulates VEGF expression and leads to a ∼27% increase in capillaries per fibre when compared to untrained individuals (Iversen et al., 2011). The background of the attenuation of VEGF expression after training in the elderly might be related to the decreases in NO^•^ bioavailability with aging. When considering the impact of physical training on the mechanisms underlying ECM remodelling and the expression of anti-angiogenic factors in the elderly there are still many unknown factors.

## Conclusions

In light of the available data, aging of the muscle–bone unit leads to capillary rarefaction, which results in limited blood flow and an impairment of skeletal muscle and bone functionality. Aging downregulates the expression of the crucial stimulators of angiogenesis, including a decrease in NO^•^ bioavailability, attenuation of the key pro-angiogenic factor VEGF and its receptors (Flt-1 and KDR), and VEGF regulator (HIF-1*α*-dependent). In contrast, habitual physical training (especially long-term cycling, jogging and walking) is an effective and helpful improvement strategy to increase capillarity in the musculoskeletal system intensifying angiogenesis in both young and older individuals. VEGF signalling pathways and VEGF regulators such as NO^•^, HIF-1*α* and PGC-1*α* are the most important factors in training-induced angiogenesis in skeletal muscles. Interestingly, as recently demonstrated, age-related changes in vital organs (liver, brain, adipose tissue, muscles and bones) were slowed down in VEGF-treated mice, which clearly demonstrates that vascular aging is a key process that influences the vitality of various organs in the body through adequate perfusion and oxygenation (Grunewald et al., 2021).

Therefore, VEGF supplementation, by enhancing VEGF signalling in muscles and bones seems to be a promising treatment strategy against age-related capillary rarefaction and impairment of tissue oxygen viability. However, one should consider that physical activity, which can be viewed as a ‘polypill’ available at low cost, covers more potential benefits to the body health, for example by attenuating the traditional CVD risk factors (*e.g.,* hyperlipidaemia, hyperglycaemia, obesity, hypertension), as well as by delaying sarco-osteoporosis. Therefore, physical activity plays an important role in slowing-down the aging process in humans (Fiuza-Luces et al., 2013; Bonewald, 2019).

## Additional Information and Declarations

### Data Availability

The following information was supplied regarding data availability: This is a review article.
